# Supplementary material for: Nurturing an organizational context that supports team-based primary mental health care: A grounded theory study
Source: PLoS One. 2024 Apr 30;19(4):e0301796. doi: 10.1371/journal.pone.0301796 (PMC11060570; doi:10.1371/journal.pone.0301796)
Supplement: S1 Table — (DOCX) [file pone.0301796.s002.docx]

### S1 Table. Interview Guide

### Background statement:

### The aim of this interview is to explore the factors that make it more or less likely for people with depression or anxiety disorders to receive high-quality care in Family Health Teams (FHTs). We will be exploring various aspects of mental health care quality and we’re interested in your point of view and experiences on these topics. The interview should last approximately 60 to 90 minutes but do not hesitate to end the interview at any time. Do you have any questions before beginning?

### Participant characteristics and intro questions:

### What is your profession and role within the FHT?

### How long have you worked at this FHT?

### How many years of experience do you have in your profession?

### Can you start by describing what care for someone with depression and anxiety looks like at this FHT?

| **Domain** | **Interview Questions** |
| --- | --- |
| **Organizational Structure** | - What organizational attributes helps or deters you and your team’s ability to provide quality mental health care for patients with depression and anxiety? - What are the incentives/disincentives to having professionals dedicated to mental health (e.g. MHW, social worker, psychologist) working full-time in the FHT? - What incentives/disincentives influence whether the team will engage in collaborative mental health care? - What does that look like here? - Communicating with one another? - Clarity of objectives? - Value of teamwork? - Transitions between different providers? - Does the FHT have an electronic medical record system? If so, how does the electronic medical record system help or hinder delivery of mental health care? - Sharing medical records across professionals and disciplines? - What are some aspects of the leadership structure that influence the provision of quality mental health care? - Are there any other organizational attributes that you can think of that might support or deter implementation of quality mental health care for this patient population? - E.g. training opportunities, regulations, norms, organizational culture & vision, access to human resources, personal beliefs, duty towards patients, stigma |
