# Supplementary material for: Nurturing an organizational context that supports team-based primary mental health care: A grounded theory study
Source: PLoS One. 2024 Apr 30;19(4):e0301796. doi: 10.1371/journal.pone.0301796 (PMC11060570; doi:10.1371/journal.pone.0301796)
Supplement: S4 File — (PDF) [file pone.0301796.s006.pdf]

Centre for Addiction
and Mental Health

camh.ca

January 30, 2019

**Study Title:**	Incentives and disincentives for treating depression and anxiety in Ontario Family Health Teams
**Principal Investigator:**	Dr. Kwame McKenzie, Centre for Addiction and Mental Health
**Co-Investigators:**	Ashcroft R, Dahrouge S, Menear M, Silveira J.
**REB #:**	140/2015
**Review Type:**	Delegated
**Approval Period:**	February 1, 2019 – February 1, 2020

Continuing Review Approval Letter

The Centre for Addiction and Mental Health Research Ethics Board (CAMH REB) has reviewed this study and has granted approval for the period noted above.

Please note the following:

- This study must be conducted as outlined in the REB approved materials, and in accordance with CAMH policies and procedures, the TCPS2 (2nd edition of the Tri-Council Policy Statement: Ethical Conduct for Research Involving Humans), the provisions of the Ontario Personal Health Information Protection Act and its applicable Regulations, and with all other applicable laws, regulations or guidelines
- No deviations from, or changes to, the protocol should be initiated without prior written approval from the CAMH REB, except when necessary to eliminate immediate hazard(s) to study participants
- Ethics approval must be renewed prior to expiry - failure to do so will result in an immediate suspension of ethics approval

REB members with a conflict of interest on a study do not participate in the discussion, deliberation or decision on such studies.

The Centre For Addiction and Mental Health Research Ethics Board operates in compliance with, and is constituted in accordance with, the requirements of the Tri-Council Policy Statement: Ethical Conduct for Research Involving Humans (TCPS 2), the International Conference on Harmonisation Good Clinical Practice Consolidated Guideline (ICH GCP), Part C, Division 5 of the Food and Drug Regulations, Part 4 of the Natural Health Products Regulations, Part 3 of the Medical Devices Regulations, and the provisions of the Ontario Personal Health Information Protection Act (PHIPA 2004) and its applicable regulations.

The CAMH REB is qualified through the CTO REB Qualification Program and is registered with the U.S. Department of Health and Human Services (DHHS) Office for Human Research Protection (OHRP).

Yours sincerely,

Dr. Robert Levitan
Chair, Research Ethics Board
Centre for Addiction and Mental Health
E-mail: Robert.levitan@camh.ca
Telephone: 416-535-8501 x 34020
RL/lb

cc: Rachelle Ashcroft <rachelle.ashcroft@utoronto.ca>; Jocelyn Booton <jocelyn.booton@utoronto.ca>
