# Supplementary material for: Nurturing an organizational context that supports team-based primary mental health care: A grounded theory study
Source: PLoS One. 2024 Apr 30;19(4):e0301796. doi: 10.1371/journal.pone.0301796 (PMC11060570; doi:10.1371/journal.pone.0301796)
Supplement: S1 File — (PDF) [file pone.0301796.s003.pdf]

33 Russell Street
Toronto, Ontario
Canada M5S 2S1
Tel: 416 535.8501

33, rue Russell
Toronto (Ontario)
Canada M5S 2S1
Tel. : 416 535.8501

www.camh.ca

camh
Centre for Addiction and Mental Health

PROTOCOL REFERENCE #140/2015-01

March 7, 2016

Dr. Kwame McKenzie
Medical Advisor
Department of Social and Epidemiological Research (SER)
Social Aetiology of Mental Illness (SAMI)
Centre for Addiction and Mental Health
80 Workman Way, Room 6302
TORONTO ON M6J 1H4

Dear Dr. McKenzie

Re: Amendment Approval for research protocol #140/2015-01 entitled "Incentives and disincentives for treating depression and anxiety in Ontario Family Health Teams" by McKenzie K, Ashcroft R, Dahrouge S, Menear M, Silveira J

We are writing to advise you that the Centre for Addiction and Mental Health Research Ethics Board (CAMH REB) has granted approval for the **amendment** to the above-named research study as described in your letter of **February 24, 2016** (Ashcroft/McKenzie).

Your revised "consent form" (Version – March 2016) **received March 7, 2016** has been approved and a copy is attached. **Subjects should receive a copy of their consent form.**

During the course of the research, any significant deviations from the approved protocol **(that is, any deviation which would lead to an increase in risk or a decrease in benefit to human subjects)** and/or any unanticipated developments within the research should be brought to the attention of the Research Ethics Office.

Best wishes for the successful completion of your project,

Susan Pilon, MHSc
Manager, Research Ethics Office,
Centre for Addiction and Mental Health

SP/ep

cc Dr. Rachelle Ashcroft; Jocelyn Booton; Jennifer Mahboob

Please retain a printed copy of this letter (and documents if applicable) for your records. Kindly quote the above reference number on any correspondence relating to this study.

FILE COPY

140/2015-01

RECEIVED
MAR - 7 2016

UNIVERSITY OF WATERLOO · RENISON UNIVERSITY COLLEGE · Bruyère · BRUYÈRE · camh · ST. JOSEPH'S · CHU de Québec · CIHR IRSC

University of Waterloo Information Letter and Consent Form

for the study entitled:

Incentives and disincentives for treating depression and anxiety in Ontario Family Health Teams

APPROVED
CAMH REB
MAR 7 2016

Principal Investigator:

Dr. Rachelle Ashcroft, PhD
Assistant Professor, School of Social Work
Renison University College, University of Waterloo
240 Westmount Road North
Waterloo, Ontario, Canada N2L 3G4

Co-Investigators:

Dr. Simone Dahrouge
Responsible Site Investigator at:
C.T. Lamont Primary Health Care Research Centre
within the Bruyere Research Institute
Ottawa, Ontario

Dr. Matthew Menear
CHU de Québec Research Centre
Université Laval
Quebec City, Quebec

Dr. Kwame McKenzie
Principal Investigator for study at:
Centre for Addiction and Mental Health
Toronto, Ontario

Dr. Jose Silveira
Local Study Lead at:
Mental Health and Addiction Program
St. Joseph's Health Centre
Toronto, Ontario

Main Contact for Study:

Jocelyn Booton
Research Coordinator for the Study at:
School of Social Work, Renison University College, University of Waterloo
240 Westmount Road North
Waterloo, Ontario, Canada N2L 3G4

Jocelyn.Booton@uwaterloo.ca
Phone: 519-888-4567 ext.28686

We are a group of researchers conducting a study investigating incentives and disincentives for treating depression and anxiety in Ontario Family Health Teams. We ask you to consider taking part in this study.

Purpose of the study

The purpose of this study is to better understand the disincentives that deter the provision of care for depression and anxiety, and identify incentives that encourage provision of treatment for depression and anxiety in Ontario's Family Health Teams.

Study participants

Researchers are conducting individual interviews with various key stakeholders who can provide insight about mental health care for common mental disorders including: Family Health Team executive directors, Family Health Team family physicians, Family Health Team nurse practitioners, Family Health Team mental health professionals and other professionals involved in mental health care, mental health providers outside of Family Health Teams, and provincial decision-makers.

Procedure

As part of the study, you will be interviewed one time for approximately 60 to 90 minutes at a place and time of your convenience. Interview questions will explore the provision of mental health care for depression and anxiety for patients of Family Health Teams.

The interview that you participate in will be audio-recorded. The recorded interview data will be transcribed and analyzed by the research team members. A copy of your transcribed interview will be emailed to you for your review. Participants are asked to read the transcript and request any changes to it within one week, after which time the transcript will be considered an accurate record of the interview that is ready to be analyzed. It is estimated that participants will take approximately 15 minutes to complete this request. We will also ask if you would be willing to be contacted at a later date in order to for us to get your opinion about our initial findings. This would involve providing your name, address, and phone number, and the name of another contact person in case you move or your phone number changes. All personal information you provide will be kept strictly confidential, separate from the interview data for the duration of the study. All computer and paper records containing your identifying information will be kept in a locked office at Renison University College at the University of Waterloo. After 15 years of completion of the study, all identifying information will be destroyed.

Risks

Although there are no physical risks and discomforts expected, you may experience psychological and emotional discomforts during interview as a result of recalling any challenges or stressful experiences you have experienced. The information you will provide will be strictly confidential, and you have a right to refuse to answer any questions that you feel uncomfortable with, or to withdraw from the study if you do not want to continue being interviewed.

Benefits

We do not expect direct benefit for you from participation in this study. However, your participation will provide information that will be useful to strengthen mental health care in Ontario Family Health Teams.

Compensation

At completion of interview, you will receive a $20 gift card as an expression of thanks for your time in this study. The amount received is taxable. It is your responsibility to report this amount for income tax purposes.

Voluntary participation

Your decision to participate in this study is voluntary. You may choose to withdraw from the study at any time even if you sign this letter of consent. Deciding not to take part or deciding to leave the study later will not result in any penalty or any loss of benefits to which you are entitled. Please contact the research coordinator, Jocelyn Booton, at jocelyn.booton@uwaterloo.ca, and detail that you want your data removed from the data set and deleted.

Rights and Confidentiality

Your identity will be kept strictly confidential to the full extent provided by law. Neither your name nor any other personal identifier will be used in any reports or publications arising from this study. All data records kept on the secured server at Renison University College at the University of Waterloo and on the researcher's computer hard disk will be password protected and accessed only by the research team members for the purpose of this study only. All study files will be stored confidentially and destroyed in 15 years. By signing this consent form, you are not waiving your legal rights or releasing the investigator(s) or involved institution(s) from their legal and professional responsibilities.

Dissemination of Results

The results of this study will be used for research presentations, conferences and publications. Should you have any questions about the study or wish to receive a copy of the results of this study, please contact either: Rachelle Ashcroft at rachelle.ashcroft@uwaterloo.ca or Jocelyn Booton at jocelyn.booton@uwaterloo.ca.

Additional Information

This study is funded by a three year grant provided by the Canadian Institutes of Health Research (CIHR), funding reference number MOP – 142435.

Statement from University of Waterloo, research host site: I would like to assure you that this study has been reviewed and received ethics clearance through a University of Waterloo Research Ethics

Committee. However, the final decision about participation is yours. If you have any comments or concerns resulting from your participation in this study, please feel free to contact Dr. Maureen Nummelin in the Office of Research Ethics at 1-519-888- 4567, Ext. 36005 or maureen.nummelin@uwaterloo.ca. This study has been reviewed by and received ethics clearance through a University of Waterloo Research Ethics Committee.

Statement from Centre for Addictions and Mental Health: As part of continuing review of the research, your study records may be assessed on behalf of the Research Ethics Board. A person from the research ethics team may contact you (if your contact information is available) to ask you questions about the research study and your consent to participate. The person assessing your file or contacting you must maintain your confidentiality to the extent permitted by law. As part of the Research Services Quality Assurance Program, this study may be monitored and/or audited by a member of the Quality Assurance Team. Your research records and CAMH records may be reviewed during which confidentiality will be maintained as per CAMH policies and extent permitted by law. Dr. Padraig Darby, Chair, Research Ethics Board, Centre for Addiction and Mental Health, may be contacted by research subjects to discuss their rights. Dr. Darby may be reached by telephone at 416-535-8501 ext. 36876.

Statement from St. Joseph's Health Centre: If you would like advice regarding your rights as a study participant, you can talk to someone who is not involved in the study at all. Please contact Dr. Hazel Markwell (Chair, Research Ethics Board) at 416-530-6750. The Research Ethics Board has reviewed the ethical aspects, physician compensation issues and financial aspects of this study and has approved it. As part of continuing review of the research, your study records may be accessed by the Research Ethics Board for verification of procedures without violating confidentiality. By signing the consent form, you are authorizing such access.

Statement from Bruyère Continuing Care: This study has been reviewed and approved by the Bruyère REB. If you have any comments or concerns resulting from your participation in this study, please feel free to contact Dr. Dorothy Kessler, Chair, Research Ethics Board, Bruyère Continuing Care at 1-613-562-6262 ext. 1420 or dkessler@bruyere.org.

The final decision about participation is yours. Your participation is important to the study. We hope you will agree to take part.

AGREEMENT TO PARTICIPATE

I, (PRINT) ______________________________, have read the Information Sheet for the study named "Incentives and disincentives for treating depression and anxiety in Ontario Family Health Teams". I understand the study procedure, the potential risks and benefits associated with my participation in the study, and my rights and confidentiality. I have had the opportunity to discuss this research study with the investigator of the research team. My questions, if any, have been answered to my satisfaction.

I understand exceptions to confidentiality in the cases that an interviewer has causes to believe:

- I am likely to harm myself and/or another person
- A child has been or may be abused or neglected

I understand that my participation in this study is voluntary and that I may choose to withdraw at any time. By signing this consent form, I do not waive any of my rights. I have been given a copy of this signed consent for my records.

Check one of the following:

☐ I **consent** to being contacted at a later time for further follow-up about the interview

☐ I **do not consent** to being contacted at a later time for further follow-up about the interview

I wish to receive a copy of my transcribed interview at the following email address: ______________________________

I agree to participate in the study.

☐ I agree to that my interview may be audio recorded for transcription and analysis

Study Participant

Name: ______________________________

Signature: ______________________________

Date: ______________________________

Person Obtaining Consent:

Name: ______________________________

Signature: ______________________________

Date: ______________________________
