# Supplementary material for: Nurturing an organizational context that supports team-based primary mental health care: A grounded theory study
Source: PLoS One. 2024 Apr 30;19(4):e0301796. doi: 10.1371/journal.pone.0301796 (PMC11060570; doi:10.1371/journal.pone.0301796)
Supplement: S2 File — (PDF) [file pone.0301796.s004.pdf]

33 Russell Street
Toronto, Ontario
Canada M5S 2S1
Tel: 416 535.8501

33, rue Russell
Toronto (Ontario)
Canada M5S 2S1
Tél. : 416 535.8501

www.camh.net

camh
Centre for Addiction and Mental Health

# PROTOCOL NUMBER # 140/2015-03

February 1, 2017

Dr. Kwame McKenzie
Medical Director, Underserved Populations Program
80 Workman Way, Room 6302
Centre for Addiction and Mental Health
Toronto, Ontario, M6J 1H4

Dear Dr. McKenzie:

**Re: Annual Renewal of protocol # 140/2015-03, entitled "Incentives and disincentives for treating depression and anxiety in Ontario Family Health Teams" by McKenzie K, Ashcroft R, Dahrouge S, Menear M, Silveira J.**

Thank you for returning the Annual Renewal of Ethics Approval form for the above-named research protocol.

We are writing to advise you that the Centre for Addiction and Mental Health Research Ethics Board has granted approval to the above-named research protocol for a period of one year.[1] Any serious Adverse Events submitted have been reviewed and acknowledged by the Research Ethics Board.
**Please keep a printed copy of this letter in your files.**

If the research is expected to continue beyond the expiry date, you are responsible for ensuring the study receives re-approval by submitting the CAMH "Annual Renewal of Ethics Approval" form by **January 1, 2018** (***please keep this date as your reminder for renewal of the study).***

During the course of research, any significant deviations from the approved protocol (**that is, any deviation which would lead to an increase in risk or a decrease in benefit to human subjects**) and/or any unanticipated developments within the research should be brought to the attention of the Research Ethics Office.

***Best wishes for the successful completion of your project.***

Susan Pilon

Susan Pilon, MHSc
Manager, Research Ethics Office
Centre for Addiction and Mental Health
SP/nz

cc : Rachelle Ashcroft <rachelle.ashcroft@utoronto.ca>

[1] CAMH Investigators are reminded that should they leave CAMH, they are required to inform the Research Ethics Board of the status of any on-going research. If a study is to be closed or transferred to another facility, the REB must be informed and any advertisements must be discontinued.
