# Supplementary material for: Nurturing an organizational context that supports team-based primary mental health care: A grounded theory study
Source: PLoS One. 2024 Apr 30;19(4):e0301796. doi: 10.1371/journal.pone.0301796 (PMC11060570; doi:10.1371/journal.pone.0301796)
Supplement: S3 File — (PDF) [file pone.0301796.s005.pdf]

UNIVERSITY OF TORONTO

OFFICE OF THE VICE-PRESIDENT,
RESEARCH AND INNOVATION

PROTOCOL REFERENCE # 33175

July 13, 2016

Dr. Rachelle Ashcroft
FACULTY OF SOCIAL WORK

Dear Dr. Ashcroft,

Re: Administrative Approval of your research protocol entitled, "Incentives and disincentives for treating depression and anxiety in Ontario family health teams"

We are writing to advise you that the Office of Research Ethics (ORE) has granted administrative approval to the above-named research protocol. The level of approval is based on the following role(s) of the University of Toronto (University), as you have identified with your submission and administered under the terms and conditions of the affiliation agreement between the University and the associated TAHSN hospital:

- Graduate Student research - hospital-based only
- Storage or analysis of De-identified Personal Information (data)

This approval does not substitute for ethics approval, which has been obtained from your hospital Research Ethics Board (REB). Please note that you do not need to submit Annual Renewals, Study Completion Reports or Amendments to the ORE unless the involvement of the University changes so that ethics review is required. Please contact the ORE to determine whether a particular change to the University's involvement requires ethics review.

Best wishes for the successful completion of your research.

Yours sincerely,

Daniel Gyewu
REB Manager

Research Oversight and Compliance Office - Human Research Ethics Program
McMurrich Building, 12 Queen's Park Crescent West, 2nd Floor, Toronto, ON M5S 1S8 Canada
Tel: +1 416 946-3273 • Fax: +1 416 946-5763 • ethics.review@utoronto.ca • http://www.research.utoronto.ca/for-researchers-administrators/ethics/
