# Supplementary material for: Nurturing an organizational context that supports team-based primary mental health care: A grounded theory study
Source: PLoS One. 2024 Apr 30;19(4):e0301796. doi: 10.1371/journal.pone.0301796 (PMC11060570; doi:10.1371/journal.pone.0301796)
Supplement: S6 File — (PDF) [file pone.0301796.s008.pdf]

33 Russell Street
Toronto, Ontario
Canada M5S 2S1
Tel: 416 535.8501

33, rue Russell
Toronto (Ontario)
Canada M5S 2S1
Tél. : 416 535.8501

www.camh.net

camh
Centre for Addiction and Mental Health

PROTOCOL NUMBER # 140/2015-03

February 1, 2018

Dr. Kwame McKenzie
Director, Health Equity
33 Russell Street
Centre for Addiction and Mental Health
Toronto, Ontario, M5A 2S1

Dear Dr. McKenzie:

Re: Annual Renewal of protocol # 140/2015-03, entitled "Incentives and disincentives for treating depression and anxiety in Ontario Family Health Teams" by McKenzie K, Ashcroft R, Dahrouge S, Menear M, Silveira J.

Thank you for returning the Annual Renewal of Ethics Approval form for the above-named research protocol.

We are writing to advise you that the Centre for Addiction and Mental Health Research Ethics Board has granted approval to the above-named research protocol for a period of one year, commencing **February 1, 2018**.[1] Any serious Adverse Events submitted have been reviewed and acknowledged by the Research Ethics Board.
Please keep a printed copy of this letter in your files.

If the research is expected to continue beyond the expiry date, you are responsible for ensuring the study receives re-approval by submitting the CAMH "Annual Renewal of Ethics Approval" form by **January 2, 2019** (***please keep this date as your reminder for renewal of the study).***

During the course of research, any significant deviations from the approved protocol (**that is, any deviation which would lead to an increase in risk or a decrease in benefit to human subjects**) and/or any unanticipated developments within the research should be brought to the attention of the Research Ethics Office.

Best wishes for the successful completion of your project.

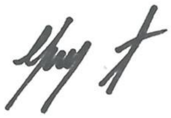

Greg Staios, MSc, CCRP
Manager, Research Ethics
Centre for Addiction and Mental Health
GS/lb

cc : Monica Emode <monica.emode@utoronto.ca>

[1] CAMH Investigators are reminded that should they leave CAMH, they are required to inform the Research Ethics Board of the status of any on-going research. If a study is to be closed or transferred to another facility, the REB must be informed and any advertisements must be discontinued.
