# Supplementary material for: Nurturing an organizational context that supports team-based primary mental health care: A grounded theory study
Source: PLoS One. 2024 Apr 30;19(4):e0301796. doi: 10.1371/journal.pone.0301796 (PMC11060570; doi:10.1371/journal.pone.0301796)
Supplement: S7 File — (PDF) [file pone.0301796.s009.pdf]

RIS Protocol Number: 37263

Approval Date: 14-Feb-19

PI Name: Dr Rachelle Ashcroft

Division Name:

Dear Dr Rachelle Ashcroft:

Re: Your TAHSN-approved research ethics protocol entitled, “Incentives and disincentives for treating depression and anxiety in Ontario Family Health Teams”

The University has conducted an Administrative review of the TAHSN REB-approved protocol titled above, and has granted approval to the attached until 2020-01-29.

Please be reminded of the following points:

- An annual Renewal must be submitted for ongoing research. Renewals should be submitted between 15 and 30 days prior to the current expiry date.
- A Protocol Completion Report (PCR) is required when research under the protocol involving the University has been completed.
- If your research is funded by a third party, please contact the assigned Research Funding Officer in Research Services to ensure that your funds are released.

Best wishes for the successful completion of your research.

OFFICE OF RESEARCH ETHICS
McMurrich Building, 12 Queen’s Park Crescent West, 2nd Floor, Toronto, ON M5S 1S8 Canada
Tel: +1 416 946-3273 ● Fax: +1 416 946-5763 ● ethics.review@utoronto.ca ● http://www.research.utoronto.ca/for-researchers-administrators/ethics
