# Supplementary material for: Nurturing an organizational context that supports team-based primary mental health care: A grounded theory study
Source: PLoS One. 2024 Apr 30;19(4):e0301796. doi: 10.1371/journal.pone.0301796 (PMC11060570; doi:10.1371/journal.pone.0301796)
Supplement: Attachment — Submitted filename: Response to Reviewers.pdf [file pone.0301796.s010.pdf]

UNIVERSITY OF TORONTO

FACTOR-INWENTASH FACULTY OF SOCIAL WORK

Dr. Johanna Pruller
Editor-in-Chief
PLOS One
January 02, 2024

Dear Dr. Pruller:

We are pleased to submit revisions to our manuscript titled "*Nurturing an Organizational Context that Supports Team-Based Primary Mental Health Care: A Grounded Theory Study*". We appreciate the reviewer's feedback and have revised the manuscript in a way that responds to all editor and reviewer comments. Please find below an itemized response to all reviewer and editor comments:

Reviewer Comments	Response
First of all, I enjoyed reading your manuscript. Overall it is a scientifically sound article. You present interesting results that may also help to improve primary mental health care in other countries.	Thank you, we appreciate your encouraging comments and also appreciate the time you took to review our manuscript.
You describe a mixed methods approach. During the first phase grounded theory was applied. Following that a semistructured interview was used. My impression is that the results from the grounded theory phase are not extensively described. Is it possible to add a short paragraph on these results (a couple of sentences)?	We added mention of grounded theory in the methods and reorganized the data collection section to enhance clarity about the grounded theory methods.<br><br>The following was added to the Data Collection section:<br>"We devised the semi-structured interview guide using sensitizing concepts that helped by providing a starting point, and consistent with grounded theory the interview questions evolved over the course of the two phases of data collection. The sensitizing concepts were derived from our previous research [46]. Interview questions explored organizational features that help and/or hinder the delivery of mental health care for patients with depression and/or anxiety (S1)."
To interpret the results it would help if the semistructured interview (or the topic list) was available as supplementary material. Is it possible to make this available (I might have overlooked)?	Interview questions are now added as a supplementary file. The following sentence was added to the data collection section:<br>"Interview questions explored organizational features that help and/or hinder the delivery of

	mental health care for patients with depression and/or anxiety (S1)."
It surprises me that so many interviews were necessary to reach data saturation. Can you elaborate a bit on this?	Thank you for raising this. We added the following to the Data Collection section to provide clarity:<br><br>"The reason why so many interviews were necessary to reach data saturation is because this study was part of a larger qualitative study investigating the financial and non-financial incentives that influence the quality of mental health care in Family Health Teams [46]. We are in process of developing a model explaining the financial and non-financial incentives which required an understanding the organizational contexts. Lastly, it required so many interviews to reach saturation because of the commitment to include the range of disciplinary perspectives that comprise this interdisciplinary team-based model of primary care."
I am not familiar with the Canadian health care system. I do know that distances in your country tend to be large, with large urban areas and more remote rural communities. I imagine that needs and availability of mental health care services may differ for these areas. Indeed, you reflect a bit on what is necessary in rural areas. Can you also reflect a bit on the needs in cities (were also teams from Toronto included?)? Do mental health care teams also hold conferences or have other meetings where they meet colleagues from other teams (as this also helps to learn from each other and improve the way care is delivered)?	We added the following sentences in Methods under the Setting sub-heading:<br>"Canada is a geographically large country comprised of ten provinces and three territories, and is sparsely populated with just over 38 million people inhabiting approximately 10 million square kilometers of land (Figure 1). With approximately 18.9% of Canadians residing in rural areas, the majority of Canadians reside in urban settings." Additionally, we added a map of Canada that illustrates Ontario.<br><br>Also, the following is now added to the results section: "There were *n* = 18 Family Health Teams represented in our sample, with *n* = 11 located in urban settings and *n* = 7 located in rural settings situated across all six of Ontario health regions (North East, North West, East, Central, Toronto, and West)."<br><br>The following was also added to the discussion section:<br>"Rural and urban primary care teams are facing various challenges in the human resources crisis.

	Geospacing analysis has demonstrated that there is an unbalanced distribution of primary care providers across the urban-rural continuum which created gaps in access even prior to the immediate crisis at hand [81]. In urban settings, however, coordinated interventions and intersectoral collaboration are needed across institutions are needed to build up the urban environment as a component of the solution [82]."
It might be interesting to add a map of Ontario with the locations of the teams that were included in the study.	We added a map of Canada that highlights Ontario. Although we agree it would be helpful to illustrate the locations of the teams, we did not add the locations to the map because it would then identify the study participants. Instead, we added the following sentence to the results section:<br><br>"There were *n* = 18 Family Health Teams represented in our sample, with *n* = 11 located in urban settings and *n* = 7 located in rural settings situated across all six of Ontario health regions (North East, North West, East, Central, Toronto, and West)."
**Editor Comments**	**Response**
Please ensure that your manuscript meets PLOS ONE's style requirements, including those for file naming. The PLOS ONE style templates can be found at<br><br>https://journals.plos.org/plosone/s/file?id=wjVg/PLOSOne_formatting_sample_main_body.pdf and<br><br>https://journals.plos.org/plosone/s/file?id=ba62/PLOSOne_formatting_sample_title_authors_affiliations.pdf	The manuscript was revised throughout to meet the PLOS ONE's style requirements including headings, references, and title page.
Please ensure that you have specified a) Did participants provide their written or verbal informed consent to participate in this study?<br><br>b) If consent was verbal, please explain i) why written consent was not obtained, ii) how you documented participant consent, and iii) whether	The following sentence was added to Data Collection section: "Informed written consent was obtained in prior to the conducting of all in-person interviews."

the ethics committees/IRB approved this consent procedure.	
In your Data Availability statement, you have not specified where the minimal data set underlying the results described in your manuscript can be found. PLOS defines a study's minimal data set as the underlying data used to reach the conclusions drawn in the manuscript and any additional data required to replicate the reported study findings in their entirety. All PLOS journals require that the minimal data set be made fully available. For more information about our data policy, please see http://journals.plos.org/plosone/s/data-availability.<br><br>Upon re-submitting your revised manuscript, please upload your study's minimal underlying data set as either Supporting Information files or to a stable, public repository and include the relevant URLs, DOIs, or accession numbers within your revised cover letter. For a list of acceptable repositories, please see http://journals.plos.org/plosone/s/data-availability#loc-recommended-repositories. Any potentially identifying patient information must be fully anonymized.<br><br>Important: If there are ethical or legal restrictions to sharing your data publicly, please explain these restrictions in detail. Please see our guidelines for more information on what we consider unacceptable restrictions to publicly sharing data: http://journals.plos.org/plosone/s/data-availability#loc-unacceptable-data-access-restrictions. Note that it is not acceptable for the authors to be the sole named individuals responsible for ensuring data access.<br><br>We will update your Data Availability statement to reflect the information you provide in your cover letter.	We are unable to publicly share our data set because although we have removed identifiable information from transcripts, we cannot remove all information that could potentially be used to identify participants. Responses to questions contain information describing Family Health Team communities, practice settings, and providers' personal roles and responsibilities, some of whom live in small rural communities where such information could be used to identify individuals. Upon reasonable request, the corresponding author is able provide de-identified data. Request for data can be submitted to Dr. David Brennan, Associate Dean of Research at the University of Toronto by email at: david.brennan@utoronto.ca
Your ethics statement should only appear in the Methods section of your manuscript. If your ethics statement is written in any section besides the Methods, please delete it from any other section.	The following was added to the Methods section of the manuscript:<br><br>"Research Ethics Board (REB) approval for this study was obtained from Bruyère Continuing Care

	(#M16–16-001), the Centre for Addiction and Mental Health (CAMH) (#140/2015), St. Joseph's Health Centre/Unity Health Toronto (#15–830), the University of Waterloo (#20973), the University of Toronto (#33175), and Université Laval (#2016–2877)."
Please review your reference list to ensure that it is complete and correct. If you have cited papers that have been retracted, please include the rationale for doing so in the manuscript text, or remove these references and replace them with relevant current references. Any changes to the reference list should be mentioned in the rebuttal letter that accompanies your revised manuscript. If you need to cite a retracted article, indicate the article's retracted status in the References list and also include a citation and full reference for the retraction notice.	The reference list was reviewed and revised. No retracted papers currently appear in the reference list.
